# Supplementary material for: Research on the dynamic changes of China’s agro-processing industry agglomeration and spatial impact of production factors on agglomeration
Source: PLoS One. 2023 Dec 22;18(12):e0292870. doi: 10.1371/journal.pone.0292870 (PMC10745219; doi:10.1371/journal.pone.0292870)
Supplement: S1 Table — (DOCX) [file pone.0292870.s001.docx]

**S1 Table .** Impact of production factor input on agglomeration of primary and deep agricultural processing industries in China

|  | **Primary Processing**  **Industries** | | **Deep Processing Industries** | |
| --- | --- | --- | --- | --- |
| **Variables** | **Main effects** | **Spatial spillover effects** | **Main effects** | **Spatial spillover effects** |
|  |  |  |  |  |
| Time-lag effect | 0.855*** |  | 0.941*** |  |
|  | (28.977) |  | (16.585) |  |
| Dual spatiotemporal lag effect | 22.153*** |  | 2.741*** |  |
|  | (106.879) |  | (8.141) |  |
| CAP | 1.288*** | -16.561*** | 0.661*** | 0.384 |
|  | (17.095) | (-40.711) | (5.503) | (0.619) |
| LAB | -2.536*** | 8.542*** | 1.264*** | 35.585*** |
|  | (-11.736) | (5.702) | (3.612) | (22.569) |
| TEC | -0.000 | 0.321*** | 0.025*** | 0.167*** |
|  | (-0.074) | (12.134) | (9.281) | (7.443) |
| GOV | 8.536*** | 83.242*** | 2.523*** | 32.230*** |
|  | (24.603) | (45.138) | (6.233) | (11.006) |
| FIN | -0.756*** | -11.324*** | -0.131** | -3.691*** |
|  | (-16.277) | (-41.412) | (-2.214) | (-11.031) |
| FDI | 0.035*** | -0.267*** | 0.041** | 0.467*** |
|  | (2.731) | (-3.316) | (2.213) | (3.764) |
| TRA | -1.951*** | -31.328*** | 0.027 | -3.860*** |
|  | (-44.571) | (-67.792) | (0.491) | (-7.933) |
| RES | 14.198*** | 44.735*** | 6.979*** | 70.885*** |
|  | (36.161) | (22.730) | (15.779) | (29.606) |
| INF | -0.040*** | -0.154*** | 0.020*** | -0.144** |
|  | (-6.053) | (-3.698) | (2.599) | (-2.258) |
| OPE | -1.205*** | -17.441*** | -0.652*** | -3.444*** |
|  | (-36.719) | (-63.304) | (-9.187) | (-10.881) |
| URB | 14.643*** | 106.380*** | 3.203*** | 25.034*** |
|  | (71.771) | (63.440) | (10.269) | (13.258) |
| Observations | 551 | 551 | 551 | 551 |
| R2 | 0.065 | 0.065 | 0.653 | 0.653 |

Note: *, **, and ***indicate significance at 10%, 5%, and 1% levels, respectively. Values in parentheses are z-statistics.
