# Supplementary material for: Research on the dynamic changes of China’s agro-processing industry agglomeration and spatial impact of production factors on agglomeration
Source: PLoS One. 2023 Dec 22;18(12):e0292870. doi: 10.1371/journal.pone.0292870 (PMC10745219; doi:10.1371/journal.pone.0292870)
Supplement: S2 Table — (DOCX) [file pone.0292870.s002.docx]

**S2 Table .** Short-term and long-term spatial effects of production factor input

on agglomeration of primary and deep processing industries.

| **Variables** | **Primary Processing Industries** | | | | | | **Deep Processing Industries** | | | | | |
| --- | --- | --- | --- | --- | --- | --- | --- | --- | --- | --- | --- | --- |
|  | Direct short-term effect | Indirect short-term effect | Total short-term effect | Direct long-term effect | Indirect long-term effect | Total long-term effect | Direct short-term effect | Indirect short-term effect | Total short-term effect | Direct long-term effect | Indirect long-term effect | Total long-term effect |
| CAP | 1.829*** | 0.267*** | 2.095*** | 0.575*** | -0.071*** | 0.504*** | 0.683*** | 0.775 | 1.457 | 1.142*** | -1.516*** | -0.374* |
|  | (24.464) | (4.601) | (37.153) | (47.966) | (-17.422) | (38.229) | (5.847) | (0.901) | (1.644) | (3.605) | (-5.076) | (-1.782) |
| LAB | -1.394*** | 0.568*** | -0.826*** | -0.344*** | 0.146*** | -0.199*** | 1.851*** | 47.555*** | 49.406*** | -8.329*** | -4.372*** | -12.701*** |
|  | (-10.146) | (3.684) | (-3.807) | (-7.362) | (11.753) | (-3.838) | (3.289) | (4.711) | (4.681) | (-7.273) | (-4.785) | (-14.682) |
| TEC | -0.029*** | -0.014*** | -0.044*** | -0.010*** | -0.000 | -0.011*** | 0.028*** | 0.230*** | 0.258*** | -0.004 | -0.062*** | -0.067*** |
|  | (-10.179) | (-4.921) | (-12.484) | (-12.172) | (-0.329) | (-12.016) | (7.932) | (4.151) | (4.432) | (-0.350) | (-7.121) | (-6.886) |
| GOV | -5.618*** | -6.984*** | -12.602*** | -2.511*** | -0.521*** | -3.032*** | 3.092*** | 43.727*** | 46.818*** | -4.873*** | -7.094*** | -11.967*** |
|  | (-4.795) | (-6.605) | (-53.049) | (-43.308) | (-26.168) | (-56.693) | (5.158) | (3.930) | (4.062) | (-2.746) | (-6.208) | (-12.429) |
| FIN | 0.860*** | 0.798*** | 1.658*** | 0.352*** | 0.047*** | 0.399*** | -0.194** | -4.959*** | -5.153*** | 0.863*** | 0.458*** | 1.321*** |
|  | (6.357) | (6.469) | (44.646) | (42.787) | (16.503) | (45.015) | (-2.403) | (-4.154) | (-4.118) | (5.008) | (3.101) | (9.622) |
| FDI | 0.032*** | -0.000 | 0.032*** | 0.009*** | -0.002*** | 0.008*** | 0.049*** | 0.616*** | 0.664*** | -0.063 | -0.111*** | -0.174*** |
|  | (3.839) | (-0.090) | (2.960) | (3.601) | (-2.614) | (2.953) | (2.951) | (3.347) | (3.554) | (-1.181) | (-3.031) | (-3.781) |
| TRA | 2.416*** | 2.150*** | 4.566*** | 0.976*** | 0.122*** | 1.098*** | -0.032 | -5.066*** | -5.098*** | 1.213*** | 0.105 | 1.318*** |
|  | (6.288) | (6.864) | (47.567) | (64.911) | (43.302) | (71.172) | (-0.404) | (-4.626) | (-4.449) | (5.493) | (0.848) | (6.881) |
| RES | -0.730 | -7.358*** | -8.088*** | -1.106*** | -0.840*** | -1.945*** | 8.207*** | 96.141*** | 104.348*** | -8.118** | -18.760*** | -26.878*** |
|  | (-0.593) | (-6.608) | (-23.650) | (-18.411) | (-34.633) | (-25.672) | (8.169) | (4.746) | (4.924) | (-2.394) | (-9.058) | (-15.262) |
| INF | 0.005 | 0.022*** | 0.027*** | 0.004*** | 0.002*** | 0.006*** | 0.018** | -0.177** | -0.158* | 0.082*** | -0.039* | 0.042* |
|  | (0.944) | (5.478) | (4.699) | (3.098) | (6.132) | (4.741) | (2.194) | (-2.125) | (-1.824) | (3.874) | (-1.933) | (1.784) |
| OPE | 1.318*** | 1.242*** | 2.559*** | 0.541*** | 0.075*** | 0.616*** | -0.713*** | -4.805*** | -5.518*** | -0.197 | 1.612*** | 1.415*** |
|  | (5.906) | (6.861) | (46.168) | (55.853) | (36.443) | (68.508) | (-7.716) | (-4.016) | (-4.418) | (-0.618) | (6.441) | (12.833) |
| URB | -6.307*** | -10.313*** | -16.620*** | -3.116*** | -0.882*** | -3.998*** | 3.626*** | 34.304*** | 37.929*** | -1.520 | -8.264*** | -9.784*** |
|  | (-3.496) | (-6.820) | (-44.747) | (-49.657) | (-62.115) | (-69.888) | (7.230) | (4.667) | (4.901) | (-0.866) | (-6.979) | (-11.294) |
| Observations |  |  | 551 |  |  | 551 |  |  | 551 |  |  | 551 |
| R2 |  |  | 0.065 |  |  | 0.065 |  |  | 0.653 |  |  | 0.653 |

Note: *, **, and ***indicate significance at 10%, 5% and 1% levels, respectively. The values in parentheses are z-statistics.
