# Supplementary material for: Research on the dynamic changes of China’s agro-processing industry agglomeration and spatial impact of production factors on agglomeration
Source: PLoS One. 2023 Dec 22;18(12):e0292870. doi: 10.1371/journal.pone.0292870 (PMC10745219; doi:10.1371/journal.pone.0292870)
Supplement: S3 Table — (DOCX) [file pone.0292870.s003.docx]

**S3 Table .** Influence of production factor input on agglomeration of agro-processing industry in China (2001–2015)

|  |  |  |  |  |  |  |  |  |
| --- | --- | --- | --- | --- | --- | --- | --- | --- |
| **Variables** | **Main effects** | **Spatial spillover effects** | **Direct short-term effect** | **Indirect short-term effect** | **Total short-term effect** | **Direct long-term effect** | **Indirect long-term effect** | **Total long-term effect** |
|  |  |  |  |  |  |  |  |  |
| Time-lag effect | 0.860***  (11.935) |  |  |  |  |  |  |  |
|  |  |  |  |  |  |  |  |  |
| Dual spatiotemporal lag effect | 0.439  (1.065) |  |  |  |  |  |  |  |
| CAP | 0.190 | -0.334 | 0.191 | -0.435 | -0.243 | 0.662 | -0.478 | 0.184 |
|  | (1.638) | (-0.665) | (1.541) | (-0.437) | (-0.230) | (0.115) | (-0.083) | (0.158) |
| LAB | 2.335*** | 59.333*** | 3.867*** | 92.193** | 96.060** | -38.547 | -74.483 | -113.030** |
|  | (8.066) | (31.391) | (2.597) | (2.463) | (2.470) | (-0.143) | (-0.271) | (-2.367) |
| TEC | 0.026*** | 0.125*** | 0.029*** | 0.208** | 0.238** | -0.016 | -0.268 | -0.284** |
|  | (8.411) | (6.560) | (7.650) | (2.311) | (2.561) | (-0.016) | (-0.271) | (-2.073) |
| GOV | 3.712*** | 52.332*** | 5.121*** | 82.758** | 87.879** | -28.421 | -74.078 | -102.500** |
|  | (11.184) | (21.964) | (3.529) | (2.351) | (2.401) | (-0.110) | (-0.282) | (-2.481) |
| FIN | -0.194*** | -5.856*** | -0.346** | -9.094** | -9.439** | 4.001 | 7.127 | 11.128** |
|  | (-3.431) | (-19.245) | (-2.073) | (-2.515) | (-2.502) | (0.150) | (0.262) | (2.341) |
| FDI | -0.048*** | -0.588*** | -0.064*** | -0.931* | -0.996** | 0.249 | 0.908 | 1.157** |
|  | (-2.977) | (-4.164) | (-3.115) | (-1.948) | (-2.017) | (0.092) | (0.324) | (2.181) |
| TRA | 0.089* | -3.145*** | 0.012 | -4.745*** | -4.734*** | 2.703 | 2.901 | 5.604** |
|  | (1.768) | (-8.024) | (0.111) | (-2.727) | (-2.584) | (0.265) | (0.274) | (2.277) |
| RES | 10.391*** | 103.175*** | 13.173*** | 163.843** | 177.016** | -47.537 | -160.548 | -208.086** |
|  | (16.008) | (35.646) | (4.735) | (2.392) | (2.485) | (-0.082) | (-0.273) | (-2.403) |
| INF | 0.014** | -0.105* | 0.012 | -0.148* | -0.136 | 0.116 | 0.056 | 0.172 |
|  | (2.082) | (-1.921) | (1.561) | (-1.654) | (-1.440) | (0.338) | (0.154) | (1.288) |
| OPE | -0.410*** | -0.112 | -0.418*** | -0.403 | -0.821* | -1.133 | 2.145 | 1.012 |
|  | (-5.625) | (-0.397) | (-5.375) | (-0.849) | (-1.656) | (-0.143) | (0.269) | (1.359) |
| URB | 3.723*** | 24.180*** | 4.382*** | 39.282** | 43.664** | -7.749 | -43.507 | -51.255** |
|  | (16.043) | (11.261) | (6.339) | (2.283) | (2.445) | (-0.048) | (-0.265) | (-2.357) |
| Observations | 406 | 406 | 406 | 406 | 406 | 406 | 406 | 406 |
| R2 | 0.053 | 0.053 | 0.053 | 0.053 | 0.053 | 0.053 | 0.053 | 0.053 |

Note: *, **, and *** indicate significance at 10%, 5%, and 1% level, respectively. Values in parentheses are z-statistics.
