# Supplementary material for: Research on the dynamic changes of China’s agro-processing industry agglomeration and spatial impact of production factors on agglomeration
Source: PLoS One. 2023 Dec 22;18(12):e0292870. doi: 10.1371/journal.pone.0292870 (PMC10745219; doi:10.1371/journal.pone.0292870)
Supplement: S4 Table — (DOCX) [file pone.0292870.s004.docx]

**S4 Table .** Impact of production factor input on average concentration ratio of China's agro-processing industry.

|  |  |  |  |  |  |  |  |  |
| --- | --- | --- | --- | --- | --- | --- | --- | --- |
| **Variables** | **Main effects** | **Spatial spillover effects** | **Direct short-term effect** | **Indirect short-term effect** | **Total short-term effect** | **Direct long-term effect** | **Indirect long-term effect** | **Total long-term effect** |
|  |  |  |  |  |  |  |  |  |
| Time-lag effect | 0.899***  (14.583) |  |  |  |  |  |  |  |
|  |  |  |  |  |  |  |  |  |
| Dual spatiotemporal lag effect | 2.547***  (8.044) |  |  |  |  |  |  |  |
| CAP | 0.634*** | -0.529 | 0.638*** | -0.400 | 0.237 | 2.590*** | -2.642*** | -0.051 |
|  | (5.782) | (-0.875) | (5.759) | (-0.437) | (0.248) | (4.688) | (-4.337) | (-0.232) |
| LAB | 1.515*** | 38.337*** | 2.465*** | 58.197*** | 60.663*** | -2.346 | -12.126*** | -14.472*** |
|  | (4.911) | (29.915) | (3.742) | (4.339) | (4.333) | (-0.830) | (-5.193) | (-16.028) |
| TEC | 0.026*** | 0.183*** | 0.031*** | 0.289*** | 0.320*** | 0.061** | -0.138*** | -0.077*** |
|  | (8.015) | (7.891) | (7.626) | (3.966) | (4.222) | (2.243) | (-5.573) | (-7.349) |
| GOV | 2.588*** | 39.242*** | 3.610*** | 60.578*** | 64.188*** | 1.800 | -17.002*** | -15.202*** |
|  | (5.861) | (14.587) | (4.386) | (3.738) | (3.800) | (0.441) | (-4.604) | (-15.971) |
| FIN | -0.117** | -3.154*** | -0.198*** | -4.841*** | -5.039*** | 0.230 | 0.965*** | 1.196*** |
|  | (-2.109) | (-10.922) | (-2.847) | (-3.701) | (-3.722) | (0.915) | (3.584) | (9.965) |
| FDI | 0.031* | 0.500*** | 0.044*** | 0.750*** | 0.794*** | 0.012 | -0.204*** | -0.193*** |
|  | (1.735) | (5.471) | (2.673) | (4.041) | (4.205) | (0.159) | (-2.987) | (-4.955) |
| TRA | -0.030 | -4.374*** | -0.135 | -6.552*** | -6.686*** | 0.825** | 0.777** | 1.602*** |
|  | (-0.453) | (-7.626) | (-1.336) | (-4.100) | (-3.987) | (2.385) | (2.295) | (6.827) |
| RES | 7.946*** | 81.489*** | 10.056*** | 126.323*** | 136.378*** | 13.582 | -46.112*** | -32.531*** |
|  | (17.983) | (27.888) | (7.102) | (4.170) | (4.307) | (1.396) | (-5.497) | (-15.943) |
| INF | 0.018*** | -0.114* | 0.016** | -0.149* | -0.133 | 0.098*** | -0.064 | 0.035 |
|  | (2.587) | (-1.866) | (2.251) | (-1.650) | (-1.420) | (2.885) | (-1.459) | (1.404) |
| OPE | -0.631*** | -3.898*** | -0.733*** | -6.165*** | -6.898*** | -1.629** | 3.272*** | 1.643*** |
|  | (-9.458) | (-11.447) | (-7.372) | (-3.797) | (-4.073) | (-2.153) | (4.774) | (12.067) |
| URB | 3.264*** | 24.254*** | 3.884*** | 38.174*** | 42.058*** | 7.493* | -17.566*** | -10.073*** |
|  | (10.371) | (11.877) | (6.854) | (4.240) | (4.447) | (1.760) | (-4.814) | (-10.450) |
| Observations | 551 | 551 | 551 | 551 | 551 | 551 | 551 | 551 |
| R2 | 0.297 | 0.297 | 0.297 | 0.297 | 0.297 | 0.297 | 0.297 | 0.297 |

Note: *, **, and *** indicate significance at 10%, 5%, and 1% level, respectively. Values in parentheses are z-statistics.
